# Thermoresponsive M1 macrophage-derived hybrid nanovesicles for improved in vivo tumor targeting

**DOI:** 10.1007/s13346-023-01378-9

**Published:** 2023-06-26

**Authors:** Antonella Barone, Anna Maria Zimbo, Nicola d’Avanzo, Anna Maria Tolomeo, Stefano Ruga, Antonio Cardamone, Christian Celia, Mariangela Scalise, Daniele Torella, Massimo La Deda, Enrico Iaccino, Donatella Paolino

**Affiliations:** 1 Department of Experimental and Clinical Medicine, University “Magna Græcia” of Catanzaro Campus Universitario-Germaneto, Viale Europa, 88100 Catanzaro, Italy; 2 Department of Cardiac, Thoracic and Vascular Science and Public Health, University of Padova, 35128 Padua, Italy; 3 Pharmacology Laboratory, Institute of Research for Food, Safety and Health IRC-FSH, Department of Health Sciences, University Magna Graecia of Catanzaro, 88100 Catanzaro, Italy; 4 Department of Pharmacy, University of Chieti – Pescara “G. d’Annunzio”, 66100 Chieti, Italy; 5 Laboratory of Drug Targets Histopathology, Institute of Cardiology, Lithuanian University of Health Sciences, A. Mickeviciaus G. 9, 44307 Kaunas, Lithuania; 6 Institute of Nanochemistry and Nanobiology, School of Environmental and Chemical Engineering, Shanghai University, Shanghai, 200444 China; 7 Department of Chemistry and Chemical Technologies, University of Calabria, 87036 Rende, Italy; 8 CNR-NANOTEC, Institute of Nanotechnology U.O.S, 87036 Cosenza, Rende Italy

**Keywords:** Extracellular vesicles, Thermoresponsive liposomes, Hybrid nanosystem, Tumor microenvironment, Macrophages

## Abstract

Despite the efforts and advances done in the last few decades, cancer still remains one of the main leading causes of death worldwide. Nanomedicine and in particular extracellular vesicles are one of the most potent tools to improve the effectiveness of anticancer therapies. In these attempts, the aim of this work is to realize a hybrid nanosystem through the fusion between the M1 macrophages-derived extracellular vesicles (EVs-M1) and thermoresponsive liposomes, in order to obtain a drug delivery system able to exploit the intrinsic tumor targeting capability of immune cells reflected on EVs and thermoresponsiveness of synthetic nanovesicles. The obtained nanocarrier has been physicochemically characterized, and the hybridization process has been validated by cytofluorimetric analysis, while the thermoresponsiveness was in vitro confirmed through the use of a fluorescent probe. Tumor targeting features of hybrid nanovesicles were in vivo investigated on melanoma-induced mice model monitoring the accumulation in tumor site through live imaging and confirmed by cytofluorimetric analysis, showing higher targeting properties of hybrid nanosystem compared to both liposomes and native EVs. These promising results confirmed the ability of this nanosystem to combine the advantages of both nanotechnologies, also highlighting their potential use as effective and safe personalized anticancer nanomedicine.

**Graphical Abstract:**

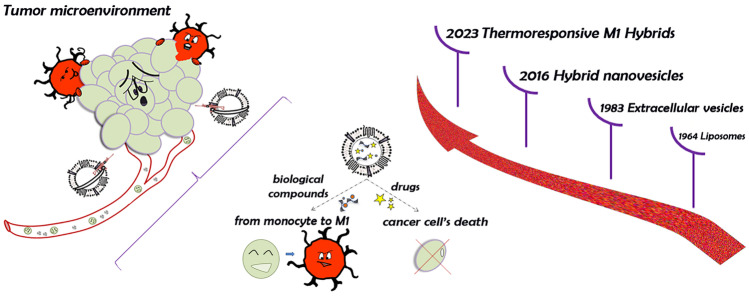

**Supplementary Information:**

The online version contains supplementary material available at 10.1007/s13346-023-01378-9.

## Introduction

Cancer plagues the world’s population with an ever-increasing incidence, and although oncological medicine is improving, its resolution appears still away for several reasons. Firstly, the failure of the healthcare systems which do not provide free access to high-quality therapies or prevention strategies, especially for low- and middle-income countries [1]. Secondly, all the oncological diseases are characterized from heterogeneity in terms of histology, pathology, pharmacological responses, and/or drugs’ resistance, which makes them highly complex systems [2].

In particular, melanoma cancer is one of the most aggressive, easy to evolve in metastasis, which affects all age populations [3]. The first-line treatment is surgery, but in case of unresectable lesions, patients need to be treated with systemic or intralesional infusion therapies [4].

In these attempts, over the years, several therapies have taken place to counter the cancer-related deaths, from common anticancer drugs to the more advanced nanotherapeutics. In this respect, nanomedicine applied to oncology was initially focused on the realization of efficacious nanosystems, especially lipid-based nanovesicles (i.e., Doxil®, Caelyx®, etc.), able to avoid most of the common side effects and increase the bioavailability of the drugs, also managing the release in a gradual and controlled manner [5, 6].

Liposomes are the most known drug delivery systems, discovered by Bangham and co-workers in 1964 [7]. Their composition and bilayer structure, so close to the cell membranes, make these systems highly biocompatible and versatile tools. This last feature is due to the easy surface functionalization, high entrapment efficiency of different drugs as well as their capability to respond to several external stimuli such as pH, magnetic field, and temperature [8–10]. In this regard, thermal responsive liposomes can increase the drug release by higher membrane permeability after induced hyperthermia [11]. The combination of physical stimuli and nanomedicine is useful for the realization of several nanoformulations for potential applications in diagnosis and therapy [12, 13].

Over time, the focus has been moved from the conventional and synthetic nanovesicles toward nanosystems of biological origin, ensuring that the main drug delivery systems’ peculiarities would be maintained. In these attempts, extracellular vesicles, previously considered just cellular communication shuttles [14], have been investigated because of their intrinsic capability for delivering genetic materials, proteins, and lipids, and even for targeting properties [15].

Considering EVs as a reflection of the parental cells, the immune cell-derived ones became a turning point in the development of anticancer nanomedicines [16]. In particular, the role of tumor microenvironment and macrophage cells is decisive in the fate of the oncological diseases [17]. The M1 phenotype indeed provides molecules as pro-inflammatory cytokines able to activate the immune system and suppress tumor during early stages [18].

Moreover, EVs can be modified in terms of loaded cargo or surface architecture. Among the engineering strategies applied, hybridization is conventionally used to increase the post-isolation loading efficiency and/or combining stimuli-responsive properties through the fusion between EVs and synthetic nanoparticles [19, 20]. Rayamajhi et al. have firstly provided a successful biomimetic nanostructure, doxorubicin-loaded, for the treatment of breast cancer. This hybrid nanosystem was made up from immuno-exosomes, collected from mouse macrophages, and synthetic liposomes [21]. Later, other hybrid nanoparticles have been proposed, in particular the thermal responsive nanovesicles, as synthetic components into the hybrids’ structure, were fused with several EVs through different techniques, such as membrane extrusion and freeze and thaw cycles [22].

On these premises, the aim of this work is to realize a generation of hybrid nanosystems, born from the fusion between M1 macrophage-derived EVs (EVs-M1) and ThermoLiposomes through the freeze and thaw method. These systems should be able to show the intrinsic capability of immune system cells to reach the tumor site, also stimulating the in situ conversion from M0 to M1 phenotype. At the same time, sharing the same liposomes’ composition, these vesicles could massively release the cargo after an induced thermal stimulus, because of the presence of phospholipids characterized by a transition temperature of ~ 42 °C. This work is mainly composed of the in vitro polarization of M1 macrophages, the isolation and purification of EVs, and ThermoLiposomes’ realization. After the hybridization process, the obtained fused vesicles have been physicochemically characterized, followed by the evaluation of the thermoresponsiveness through in vitro fluorescent probe release. Moreover, the assessment of the tumor targeting properties has been evaluated through in vivo animal studies, in which melanoma cells have been implanted into mice in order to develop a solid tumor mass. The purpose of this detailed characterization is laying the foundation for the development of a nanoplatform in which combining the advantages of liposomes and EVs-M1, for potential personalized chemo-immune anticancer therapies.

## Materials and methods

### Materials

1,2-dipalmitoyl-sn-glycero-3-phospocholine (DPPC), 1-myristoyl-2-stearoyl-sn-glycero-3-phosphocholine (MSPC), and 1,2-distearoyl-sn-glycero-3-phosphocholine (DSPC) were purchased from Avanti Polar Lipids Inc. (Birmingham, AL, USA). Lissamine Rhodamine B 1,2-dihexadecanoyl-sn-glycero-3 phosphoethanolamine (rhodamine-DHPE) was provided by ThermoFisher Scientific Co., Ltd. (USA). Disodium fluorescein was obtained from Sigma-Aldrich Co. (St. Louis, MO, USA). Lipopolysaccharide (LPS) was provided by InvivoGen (San Diego, CA, USA), and Interferon gamma (INF-γ) was purchased from Miltenyi Biotec (Bergisch Gladbach, Germany). The cell line J774A.1 was obtained from American Type Culture Collection (ATCC, USA). Fetal bovine serum (FBS) and exosome-depleted FBS were provided by Thermo Scientific (Paisley, UK). All the other reagents used in the experiments were of analytical grade (> 98%).

### Methods

#### Thermo-responsive liposomes realization

The liposome suspension was realized through the thin layer evaporation method (TLE), as previously reported by Lv et al. with some modifications [23]. The lipid mixture was composed of DPPC:MSPC:DSPC in the molar ratio 86:10:4, respectively. It was dissolved in an organic solvent solution (chloroform/methanol, 3:1 v/v) and evaporated through rotavapor Büchi R-210 at 45 °C. Thus, the obtained lipid film was hydrated with PBS (0.01 M, pH 7.4) in a final lipids’ concentration of 20 mg/mL. Then, this suspension was subjected to three cycles of warming at 45 °C in a water bath and vortex at 750 rpm; finally, the colloidal formulation was left to stabilize at 45 °C for 2 h. In order to obtain small unilamellar liposomes, scalar extrusions through polycarbonate membrane filters from 400 to 100 nm were done, by using a Lipex Biomembranes extruder (Northern Lipids Inc., Vancouver, BC, Canada) at 45 °C. When required, Rhodamine-DHPE (0.1% mol/mol) was co-dissolved with the starting lipids mixture.

The fluorescent probe (disodium fluorescein, 1 mg/mL) used for in vitro thermo-responsiveness investigation was solubilized into the hydrophilic phase during the liposome realization.

#### In vitro macrophage culture and polarization

J774.A1 murine macrophage cells were employed for these studies. In particular, 8.5 × 10^5^ cells were seeded per vented T75 flask and cultured with Dulbecco’s Modified Eagle’s Medium (DMEM) (Sigma Chemical Company, St. Louis, MO, USA) supplemented with 10% v/v FBS, 1% v/v pen/strep, and 1% v/v L˗glutamine. In order to induce the M1 phenotype, 1 day post seeding, cells were treated with 10 ng/mL LPS and 50 ng/mL INF˗γ and incubated for 16 h [24]. Then, the M1 polarization was validated through light microscopy and flow cytometry analysis through the evaluation of CD86 marker expression [25].

#### Extracellular vesicles isolation and purification

The medium of both M0- (non-induced control) and M1-like macrophages was replaced with fresh D˗MEM, supplemented with 10% v/v exosome-depleted FBS for 48 h, before culture-conditioned medium (CCM) collection. The collected CCM was previously centrifuged twice at 700 × g for 5 min and 2000 × g for 10 min at 4 °C and then filtered through a 0.22 μm filter unit, in order to remove debris and detached cells [26]. Later, the starting volume was concentrated by using Centricon Plus-70 centrifugal filters (Millipore Sigma) up to a final volume of 1 mL. The samples’ purification was performed through size exclusion chromatography (SEC) with qEV original 70 nm columns (Izon Science, Cambridge, MA). All the columns were rinsed with PBS, and then 1 mL of each sample was loaded onto each column, followed by fractions’ extraction. Finally, the recovered volume was concentrated by using 50 kDa MWCO Amicon Ultra-15 centrifugal filters up to a final volume of 1 mL [27]. When required, fluorescent EVs were labeled after isolation by 1 h of incubation at 37 °C with carboxyfluorescin diacetate succinimidyl ester (CFDA-SE). This latest, after passing the membranes, undergoes hydrolysis of the diacetic groups, thus coverting into the fluorescent state CFSE [28, 29]. The fluorophore excess was removed by the use of centrifugal filters.

#### Hybridization process

The hybrid formulations were realized through the freeze and thaw technique [23]. Briefly, rhodamine-DHPE ThermoLipo and CFSE-labeled EVs, in the number ratio 1:1 (1 × 10^10^ vesicles/mL), were fused through 10 total cycles: 5 min in liquid nitrogen and 15 min in a water bath at 37 °C. The resulting dual-marked hybrid nanovesicles were used for in vivo imaging studies and ex-vivo FACS analysis.

Conversely, for in vitro thermoresponsive evaluation, the fluorescein-loaded hybrid nanovesicles were obtained by fusing disodium fluorescein-loaded liposomes and native EVs, both without the presence of rhodamine DHPE lipid and CFSE, respectively, in order to prevent the overlapping phenomena. The release kinetic profiles of fluorescein were then studied as below reported in the “In vitro thermoresponsiveness” section.

#### Physicochemical characterization

The main physicochemical characteristics such as hydrodynamic diameter, polydispersity index (PdI), zeta potential (ZP), and particle concentration were assessed at 25 °C through Zetasizer Ultra (Malvern Instruments Ltd, Malvern, UK), by using a backscattering detection angle of 173°. ZP values were obtained referring to the Helmholtz-Smoluchowski equation for the electrophoretic mobility, after a proper aqueous dilution of the samples.

The emission spectrum of fluorescein-loaded liposomes was recorded in a wavelength range between 500 and 600 nm after excitation at *λ*_ex_ = 490 nm.

The long-term stability features were investigated through Turbiscan Lab® Expert (Formulaction, L’Union, France) in terms of light transmitted (ΔT) and backscattered (ΔBS), by following all the parameters previously reported [30]. Moreover, turbiscan stability index (TSI) values were considered in order to outline the destabilization kinetic profiles. All the samples were diluted 1:10 (final volume 10 mL) in isosmotic buffer. The analyses were performed for 1 h at 25 °C.

TEM analyses were performed as previously described by Palmosi et al. [31]. Briefly, the samples were diluted ~ 200 times with an inert and isotonic buffer and then placed on a formvar-coated 200-mesh copper grid (TABB Laboratories Equipment, UK). Nanovesicles were stained by using uranyl acetate solution (2% w/v) and then dried overnight at room temperature. Images’ acquisition was performed through Tecnai G2 (FEI) transmission electron microscope (TEM) operating at 100 kV. Images were captured with a Veleta (Olympus Soft Imaging System) digital camera.

Total protein contents of M0 and M1 EVs, as well as hybrid systems, were quantified by using BCA Protein Assay according to the manufacturer’s instructions (MicroBCA™ Protein Kit, Life Technologies, 23,235). The resulting data were carried out by using BSA external calibration curve, in the concentration range of 0.1–2 mg/mL.

#### FRET assay

Steady-state emission spectra were recorded by using HORIBA Jobin–Yvon Fluorolog-3 FL3-211 spectrometer equipped with a 450 W xenon arc lamp, double-grating excitation and single-grating emission monochromators (2.1 nm/mm dispersion; 1200 grooves/mm), and a Hamamatsu R928 photomultiplier tube. The emission and excitation spectra were corrected for source intensity (lamp and grating) and emission spectral response (detector and grating) by standard correction curves. The potential FRET/quenching effect was evaluated by overlapping the emission spectra of CFSE-EVs, hybrid nanovesicles (double-labeled nanovesicles), and a physical mixture of CFSE-EVs and rhodamine DHPE-liposomes (Rho-Lipo). Then, the results were evaluated by following the intensity of emission spectra at the maximum CFSE emission wavelength (*λ* = 525 nm).

#### Cytofluorimetric analysis

For the validation of macrophage polarization, the antibody, anti-mouse CD86 (FITC) (cat. no. 11–0860-82, eBioscience), was incubated with cells for 45 min at 4 °C and washed three times with fresh PBS to remove the excess.

The happened liposomes-EVs hybridization was validated in order to check the co-localization of Rhodamine-DHPE lipid and anti-CD63-APC. Specifically, vesicles were conjugated with CD63-coupled magnetic beads provided by SBI’s Exo-Flow IP kit (SBI) according to the manufacturer’s instructions. Immunoconjugates were then stained with anti-CD63-APC (cat. No. 130–108-894, Miltenyi Biotec). The markers’ expression was then quantified through FACSCanto II flowcytometer (Becton Dickinson, San Jose, CA, USA) and analyzed with FlowJo software (Tree Star, Inc., Ashland, OR, USA).

#### In vitro thermoresponsiveness

Thermoresponsive behavior was assessed through in vitro release studies of fluorescein. Briefly, 1 mL of fluorescein-loaded ThermoLipo or hybrid nanosystems, previously purified, was put into a dialysis bag (cut off 10 kDa) and then in 100 mL of PBS (0.01 M, pH 7.4) under continuous magnetic stirring at 37 °C and 42 °C. Afterward, 1 mL of dialysis medium was collected at 2, 5, 10, 15, 20, 30, 45, and 60 min, and the volume withdrawn was soon replaced with fresh solution. The fluorescent probe was determined by using fluorimetric analysis, through spectrometer LS55 (Perkin-Elmer). The obtained results were acquired with FL WINLAB tm software, and the quantification of released fluorescein was referred to an external calibration curve with a concentration range of interest from 0.0025 to 0.1 µg/mL.

#### Animal

Male C57BL/6 mice (22–24-week-old, 20–25 g) were obtained from Charles River Laboratories. All animal experimental protocols were approved by the Bioethical Committee of the University Magna Graecia of Catanzaro, even in accordance with the protocol n.794/2016-PR approved by the Italian Ministry of Health. Mice were housed under controlled environmental conditions (25 °C and 50% relative humidity) with a 12 h light/dark cycle. Eighteen animals were employed: 3 animals for each group.

#### In vivo tumor induction and experimental groups’ design

B16F10 murine melanoma cell suspension (1 × 10^6^ cells) in a volume of 0.2 mL of PBS was injected subcutaneously under the right shoulder blade of mice [32]. The tumor mass was let it grow for 10 days in order to develop an appreciable tumor mass. A day later, each group was treated with 0.2 mL of PBS (control) or dispersions of ThermoLipo, M0 EVs, M1 EVs, Hybrid M0, and Hybrid M1, respectively, via tail vein injection.

#### In vivo TME targeting

The TME targeting capability of nanosystems was evaluated through in vivo imaging with the Bruker In-Vivo Xtreme X-ray/optical imaging system at 1 h and 3 h post-treatment injection, by using Bruker MI software (Bruker, Billerica, MA) for images acquisition and analysis [33]. In order to proceed with the acquisition, animals were anesthetized with 4% isoflurane and placed into the optical imaging chamber, continuously ventilated with 2.5% isoflurane and 2% oxygen, keeping the animal bodies temperature at 37 °C. The acquisition channels were fixed at emission wavelengths of 590 nm and 520 nm, in order to detect Rhodamine-DHPE and CFSE dye, respectively.

#### Tumor tissues dissociation and analysis

After 3 h-images acquisition, mice were sacrificed, and the tumor mass tissues were mechanically strained with 70 and 40 µm cell strainers prior to further investigation with FACS analysis.

### Statistical analysis

Significant differences among data were evaluated by one-way analysis of variance (ANOVA) and Tukey’s multiple comparison test. The analyses were performed by using Excell and Sigmaplot software. Three different significant levels have been used *p < 0.05, **p < 0.01, and ***p < 0.001.

## Results and discussion

### In vitro macrophage polarization and extracellular vesicles purification

The immune system acts as the main player in several pathologies, including cancer [34]. In particular, tumor-associated macrophages (TAMs) are key elements in TME, which can reach 50% of the tumor mass, deeply affecting cancer behavior. Macrophage derived from circulating monocytes can be divided into two main categories: M1-macrophages (also known as classically activated) with tumor-suppressing function and M2-macrophages (also known as alternatively activated) with pro-oncogenic features [35]. Due to the inflammation in TME, as well as the presence of several M2-inducing factors, i.e., IL-4, IL-10, TGF-β, TAMs are overbalanced toward M2-phenotype, attracting the attention of the scientific community as a target for the development of innovative anticancer therapies [36]. In these attempts, two main approaches have been investigated: the reduction of TAMs and their re-education toward the M1-phenotype [37–39]. Recently, M1 macrophage-derived EVs have been studied for this purpose, demonstrating suitable tumor homing properties and the ability to promote the repolarization from M2 to M1 macrophage phenotype in vitro and in vivo [40, 41].

In this study, the murine macrophage cell line J774A.1 was investigated, and in particular the non-induced phenotype M0, as a negative control, and the induced M1, both as extracellular vesicles’ source. The importance of these vesicles consists of their capability to reflect the same superficial architecture of the parental cells [42]. After inflammatory cascade activation, the immune system cells are attracted by an inflamed site and/or tumor microenvironment, in which different adhesion molecules such as selectins and integrins play as mediators between leukocytes and tissues [43].

**Fig. 1:**
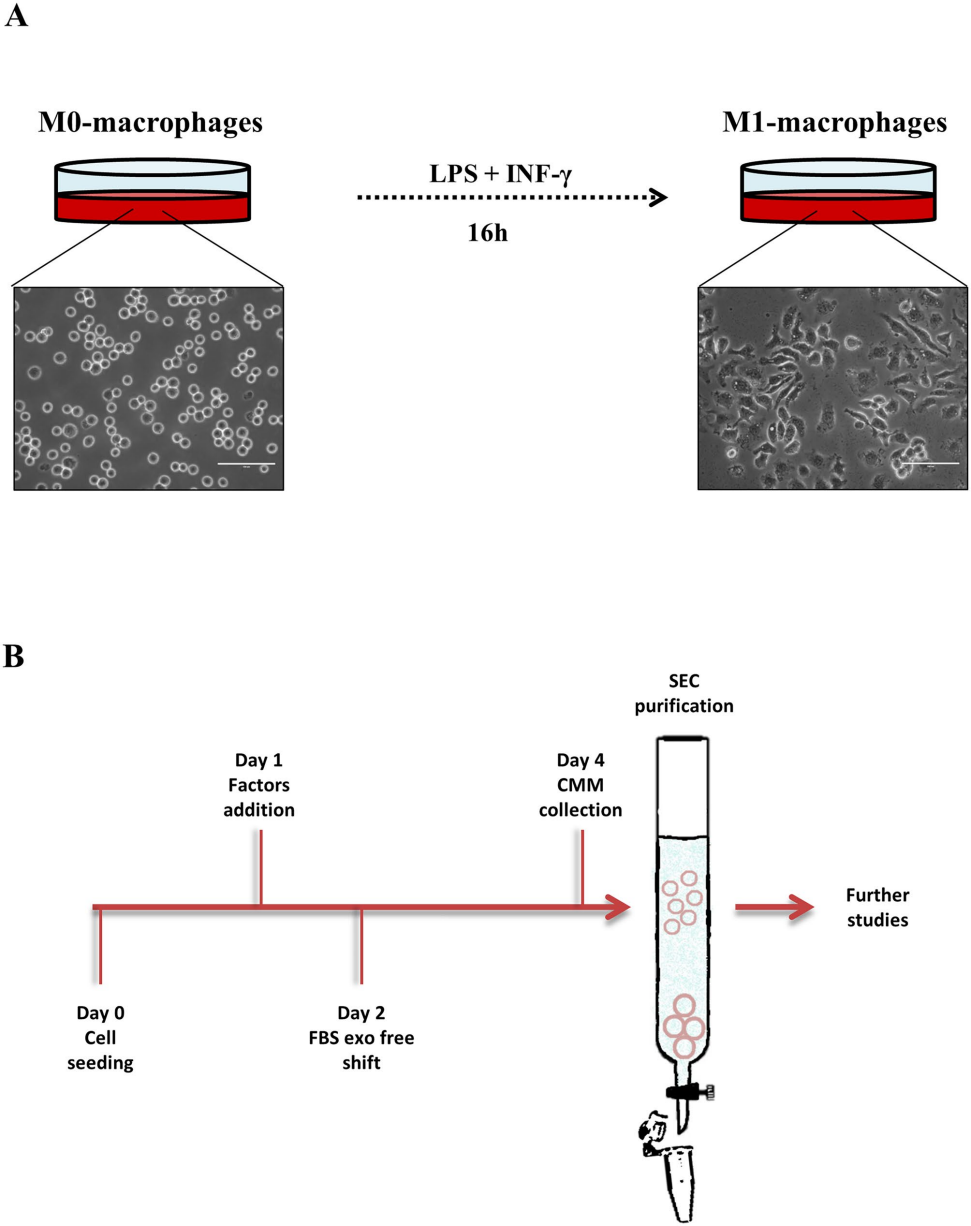
EVs’ sources and purification scheme. **A** shows representative light microscopy photograms of M0 and M1 macrophages. **B** is a schematic representation of extracellular vesicles isolation design

The phenotype M1 was activated by the treatment with LPS and IFN-γ, which results in a metabolic shift from oxidative phosphorylation toward a glycolytic anaerobic pathway in order to cope with both an increasing demand of energy and pro-inflammatory proteins’ precursors production [44]. Moreover, as Fig. 1A highlights, the non-induced M0 macrophages showed a typical round shape, distinctly different from the M1 fusiform shape [45]. The polarization was further studied by FACS analysis, investigating the presence of biomarker CD86 [46]. As reported in Fig. S1 (ESM_1), after the induction of the M1 phenotype, macrophages showed a shift toward the CD86 marker.

The nanomedicine research field has been evolving, also putting a greater focus on therapeutic nanosystems’ features and the development of personalized therapies through different strategies [20, 47]. In this regard, extracellular vesicles, firstly considered just cellular messengers, were later focused as drug delivery systems because their structure and composition are similar to the conventional and synthetic nanovesicles commonly used in nanomedicine, displaying at the same time unique properties in terms of targeting, payloads, and surface architecture.

Extracellular vesicles were collected from the conditioned media (D-MEM supplemented with 10% v/v FBS exo-free) incubated for 48 h and then purified through size exclusion chromatography by separating particles and molecules which eluate through the resin pores at different times, according to their size [48], as shown in Fig. 1B.

### EVs-ThermoLipo hybridization

Although EVs can be considered as natural and efficient drug delivery systems per se [49], several key points should be addressed, such as the low entrapment efficiency and/or the possibility to modify the cargo release. On this basis, hybrid nanosystems have been realized in order to compensate for all the limitations of both liposomes and EVs, by conjugating these latest in the form of biomimetic and semisynthetic vesicles [50].

Hybrid nanosystems have been realized through 10 cycles of freezing in liquid nitrogen and thawing at 37 °C, a method already known for both liposomes preparation [51] and hybridization between liposomes and extracellular vesicles, modifying some parameters [52]. This process allows a temporary break into the lipid bilayer, followed by the thawing step in which the proper temperature provides a high fusion efficiency [19].

**Fig. 2:**
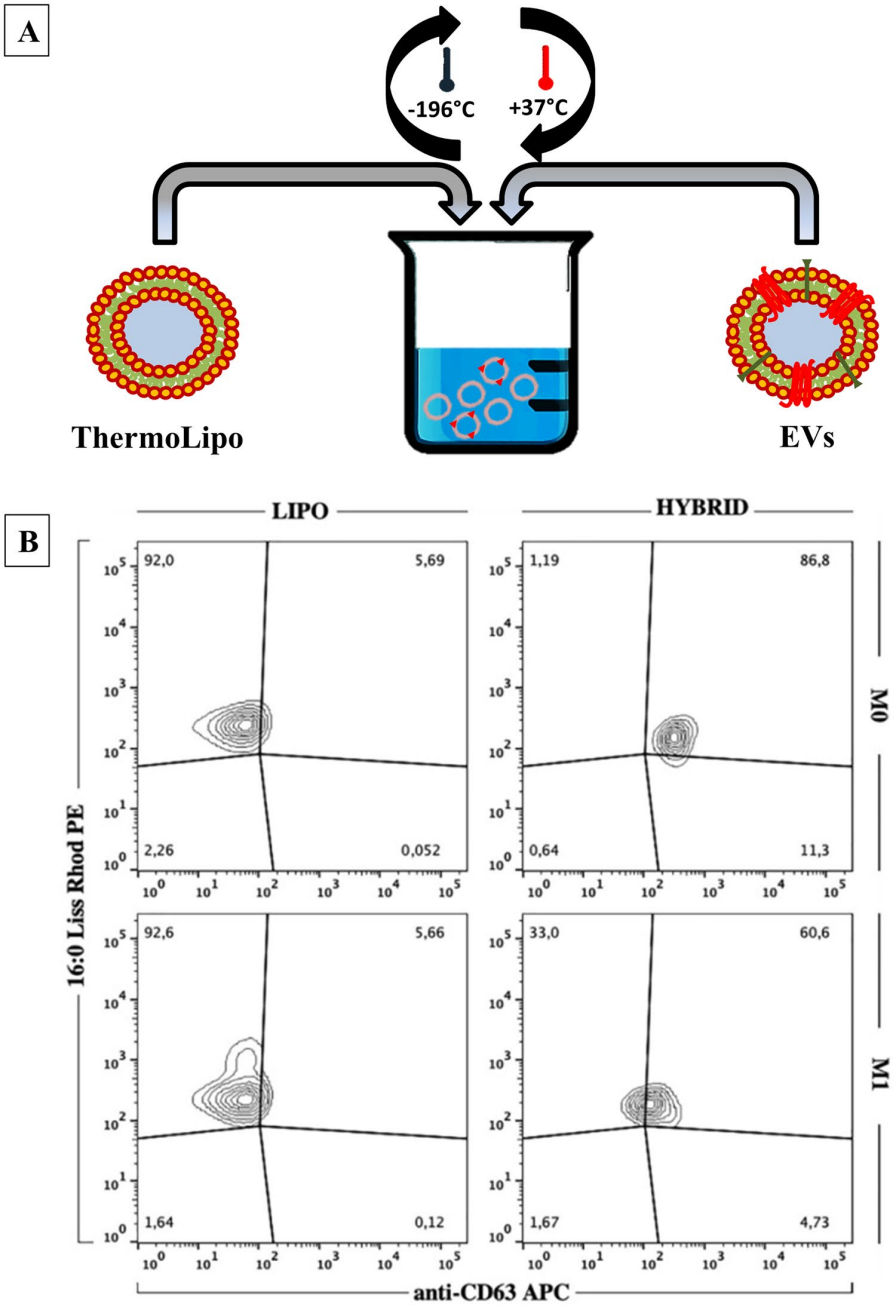
Hybridization evaluation. **A** is a schematic representation of hybridization process, while **B** reports the cytofluorimetric analysis of hybrid nanosystems. Results are representative of three independent experiments

The happened hybridization has been verified by the use of FACS analysis (Fig. 2), able to show the co-localization of both Rhodamine-DHPE placed into the liposomes’ bilayer and anti-CD63-APC positive population [53]. As Fig. 2 shows, almost all the CD63-sorted vesicles are also PE-positive, demonstrating that the fusion process worked properly. Moreover, focusing carefully on the FACS plots, it is important to highlight the integrity of the vesicles’ membrane after the freeze and thaw process, demonstrated by the presence of CD63. Therefore, this analysis leads to the conclusion that the detection of CD63 protein, after the isolation and hybridization process, may be considered as an indirect proof of the validation process.

### Physicochemical characterization

Liposomes can be considered as the most known nanosystems and among the first ones to reach the market, because of their efficacy, safety, and system’s biocompatibility [54, 55]. Furthermore, their capability to act under different exogenous stimuli, based on the phospholipids features, makes them highly versatile [56].

In this respect, the thermoresponsive formulation was realized by using phospholipids with low sol–gel transition temperature, which reflects in temperature-responsive conformational changes in the bilayer structure, thus increasing its flexibility and permeability [11]. ThermoLipo were realized by using a lipid composition similar to ThermoDox^®^, a formulation currently under phase I for the treatment of different solid tumors, i.e., pancreatic, pediatric refractory, and metastatic breast cancer. The absence of cholesterol in this formulation is crucial to guarantee a rapid and narrow transition temperature of lipid conformation under mild hyperthermia (42 °C) [57]. Moreover, based on the aim of this work, PEGylated lipids were not used to avoid the hampering of potential tumor-targeting properties of resulting hybrid nanosystems as well as the reduction of hybridization rate, due to the stealth features of PEG. This approach was in line with other studies involving the use of leukocytes-mimetic nanovesicles [58].

**Fig. 3:**
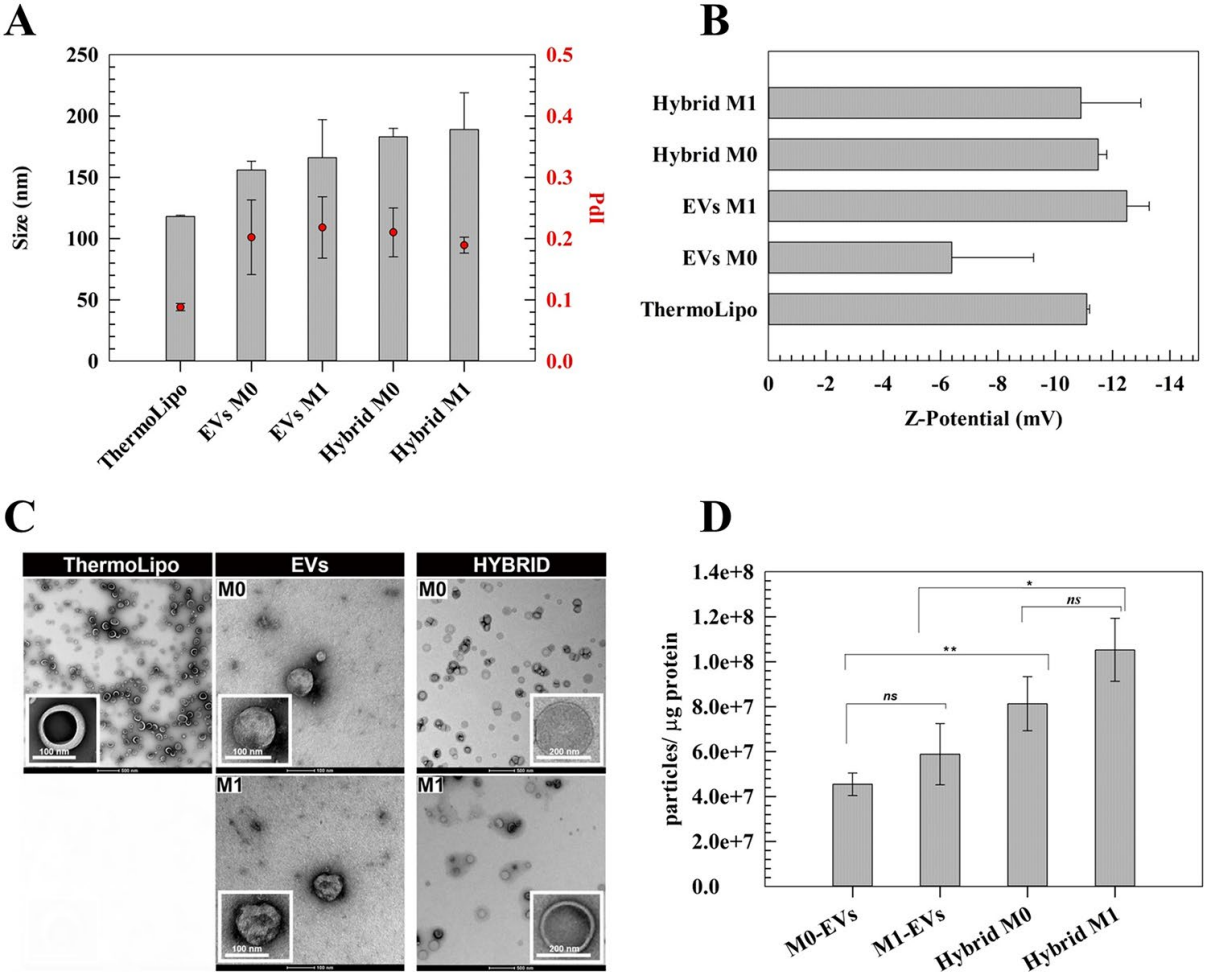
Physicochemical characterization of ThermoLipo, EVs, and hybrid nanovesicles. **A** shows the average size and PdI of nanovesicles, and **B** is the zeta potentials of nanovesicles. **C** represents transmission electron microscopy (TEM) with two different scale bars: ThermoLipos’ and EVs’ higher-magnification images 100 nm and hybrids’ higher-magnification images 200 nm. **D** represents total proteins recovered from each sample determined by MicroBCA protein assay. Results are expressed as ratio between the particle concentration and protein content (μg). Data are the average of three independent analyses ± S.D

ThermoLipo were realized through the TLE technique and physicochemically analyzed through DLS. The mean size obtained (118 ± 1 nm) was also confirmed by TEM images, which showed a round shape of nanovesicles (Fig. 3C). The presence of the fluorescein probe did not affect the systems, showing no significant changes compared to empty liposomes (Table S1). The size distribution (PdI) (Fig. 3A) (0.088 ± 0.006) and zeta potential value (− 11.1 ± 0.1 mV) (Fig. 3B) also confirm a high monodispersity as well as moderate colloidal stability [59].

M0- and M1-derived EVs were also investigated with DLS, showing hydrodynamic diameter, PdI, and zeta potential values (Fig. 3A, B) in line with the common EVs isolated from CCM [21]. In particular, no significant difference in terms of average diameter has been recorded for M0-EVs and M1-EVs which showed an average diameter of 156 ± 7 nm and 166 ± 31 nm, respectively. Conversely, slight difference between M0-EVs and M1-EVs was observed for zeta potential (− 6.39 ± 2.86 vs − 12.5 ± 0.78), thus reflecting a different surface architecture of donor cells (Fig. 3A, B). Also for EVs, their labeling did not significantly modify their physicochemical properties (Table S1).

ThermoLipo and EVs were then employed for the realization of hybrid nanovesicles by freeze and thaw technique, and the resulting nanosystems demonstrated an average diameter below than 200 nm. Noteworthy, the hybridization procedure reduced the differences in terms of zeta potential between the two formulations, thus showing a “leveling effect.” All hybridized/isolated nanovesicles demonstrated an average diameter below than 200 nm and a PdI value below than 0.3, thus showing suitable physicochemical properties for a potential in vivo administration [60].

DLS data was also confirmed by TEM that demonstrated a round shape for all natural and semisynthetic nanovesicles (Fig. 3A, C).

Moreover, the total proteins of bio-derived nanovesicles were analyzed and reported as a ratio between particle concentration and protein content (Fig. 3D). Since EVs were diluted 1:1 ratio for the hybridization, the total amount of recovered proteins in M0- and M1-derived EVs are about twice compared to the protein contents in the resulting hybrid nanosystems, considering the same starting number of particles. No statistical differences are reported between M0-EVs and M1-EVs, thus assuming the optimization of the isolation process. Similar results were obtained by comparing each other M0- and M1-hybrids, so demonstrating no damages/loss during the freeze and thaw.

### FRET assay

Fluorescence resonance energy transfer (FRET) is a well-known mechanism which describes the energy transfer between a donor and an acceptor chromophore, and it is widely used to study membrane fusion processes [61, 62]. This mechanism is highly dependent on several factors, like the distance between the donor and acceptor chromophores and their mol ratio [21]. The occurrence of the FRET effect leads to a consistent decrease of intensity (or even quenching) of donor chromophore emission and an increase of emission intensity in the acceptor chromophore.

To study the presence or lack of FRET effect in the hybrid nanovesicles, the steady-state photophysical properties (i.e., emission and excitation spectra) were compared to CFSE-EVs and the physical mixture of CFSE-EVs and Rho-Lipo. We first studied the maximum excitation/emission wavelengths of CFSE-EVs and Rho-Lipo (Fig. S3), showing a *λ*_ex/em_ of 490/525 nm for CFSE-EVs and 570/590 nm for Rho-Lipo, respectively.

**Fig. 4:**
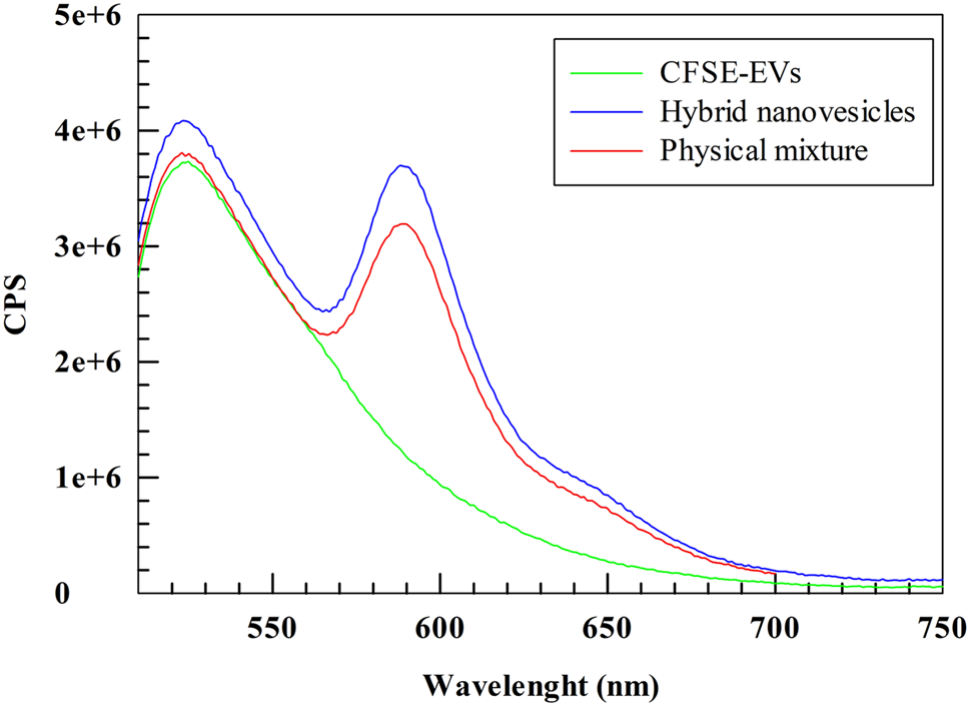
FRET study. The emission spectra of CFSE-EVs, physical mixture of CFSE-EVs + Rho-Lipo, and hybrid nanovesicles were recorded (*λ*_ex_ = 490 nm)

Moreover, the potential occurrence of the FRET effect, all the samples were then excited at 490 nm (*λ*_ex max_ of CFSE), and the resulting emission spectra were recorded from 510 to 750 nm. No significant reduction of CFSE emission intensity was recorded at 525 nm in all the tested samples (Fig. 4). This result demonstrated that the FRET did not occur for hybrid nanovesicles. Interestingly, a slight increase in the emission intensity for both chromophores was recorded for hybrid nanovesicles during the analysis. We supposed that the resulting increased intensity for both chromophores in hybrid nanovesicles may depend on the different refractive index of this sample and/or the rearrangement of the supramolecular structure of nanovesicles during the hybridization process.

### Long-term stability studies

**Fig. 5:**
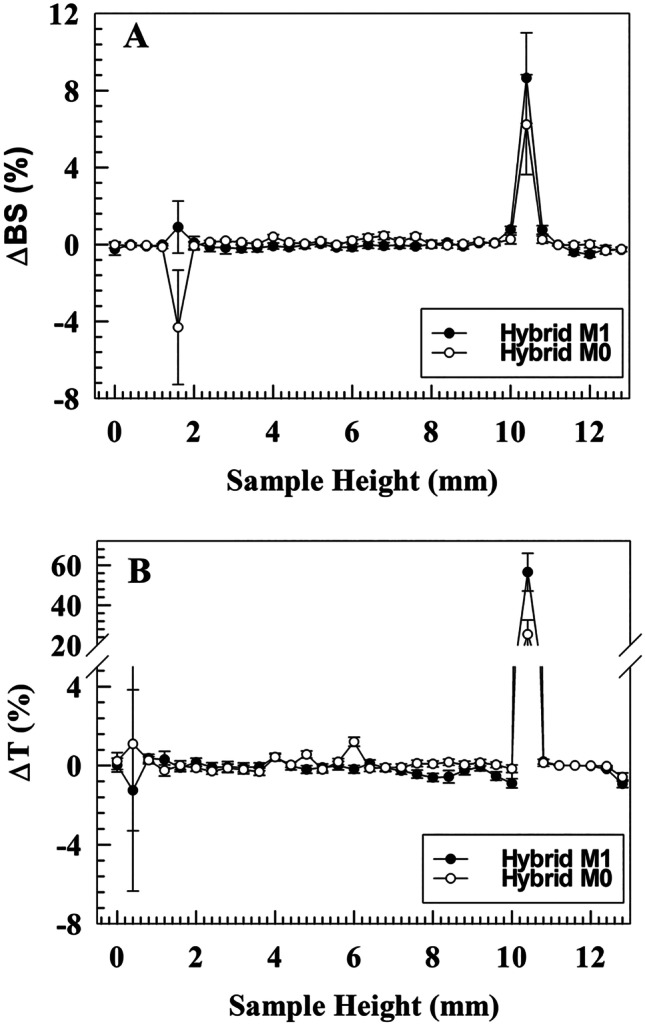
Turbiscan analysis of hybrid nanosystems. **A** Δ Back scattering (45° angle); **B** Δ Transmittance (180° angle). Results are the mean of three independent analyses ± S.D

In order to predict the long-term stability, resulting hybrid nanosystems were investigated through the Turbiscan Lab® Expert instrument. The outcomes (Fig. 5) have shown no variations above 5% for ΔBS and ΔT values, considering the sample’s height between 2 and 8 mm. Heights upper or lower than this range are affected by the presence of air bubbles at the liquid–air interface and at the bottom of the vial, respectively [63]. These findings showed the absence of destabilization phenomena such as flocculation, sedimentation, and/or creaming. The obtained data were also in agreement with the destabilization kinetic studies (TSI), demonstrating profiles in line with other nanovesicles realized by our group [30] and suggesting suitable physical stability (Fig. S4, ESM_1). Noteworthy, TSI analysis demonstrated that the hybridization process stabilizes the resulting hybrid nanovesicles regardless of the donor source. Although these findings are still not completely clear and need more investigations, they seem to suggest the instauration of constructive interactions between semisynthetic lipids derived from liposomes and lipids/proteins derived from EVs (Fig. S4, ESM_1). This “stabilization effect” was recorded for both hybrid nanovesicles, and it was more evident for hybrid M1, highlighting the preservation of peculiar features based on the cellular source.

### In vitro thermo-responsiveness evaluation

Temperature represents one of the physical parameters capable of modifying the cargo’s release from the thermoresponsive formulations, improving their efficacy. The advantage in the use of this kind of system began as a strategy to exploit local hyperthermia induced through several processes, such as near-infrared radiation of a specific tissue. Basically, this method is able to ablate the proliferative response of the tumor cells, but also, in presence of thermoresponsive lipid-based nanotherapeutics, to affect their transition temperature and increase their membrane permeability [64].

**Fig. 6:**
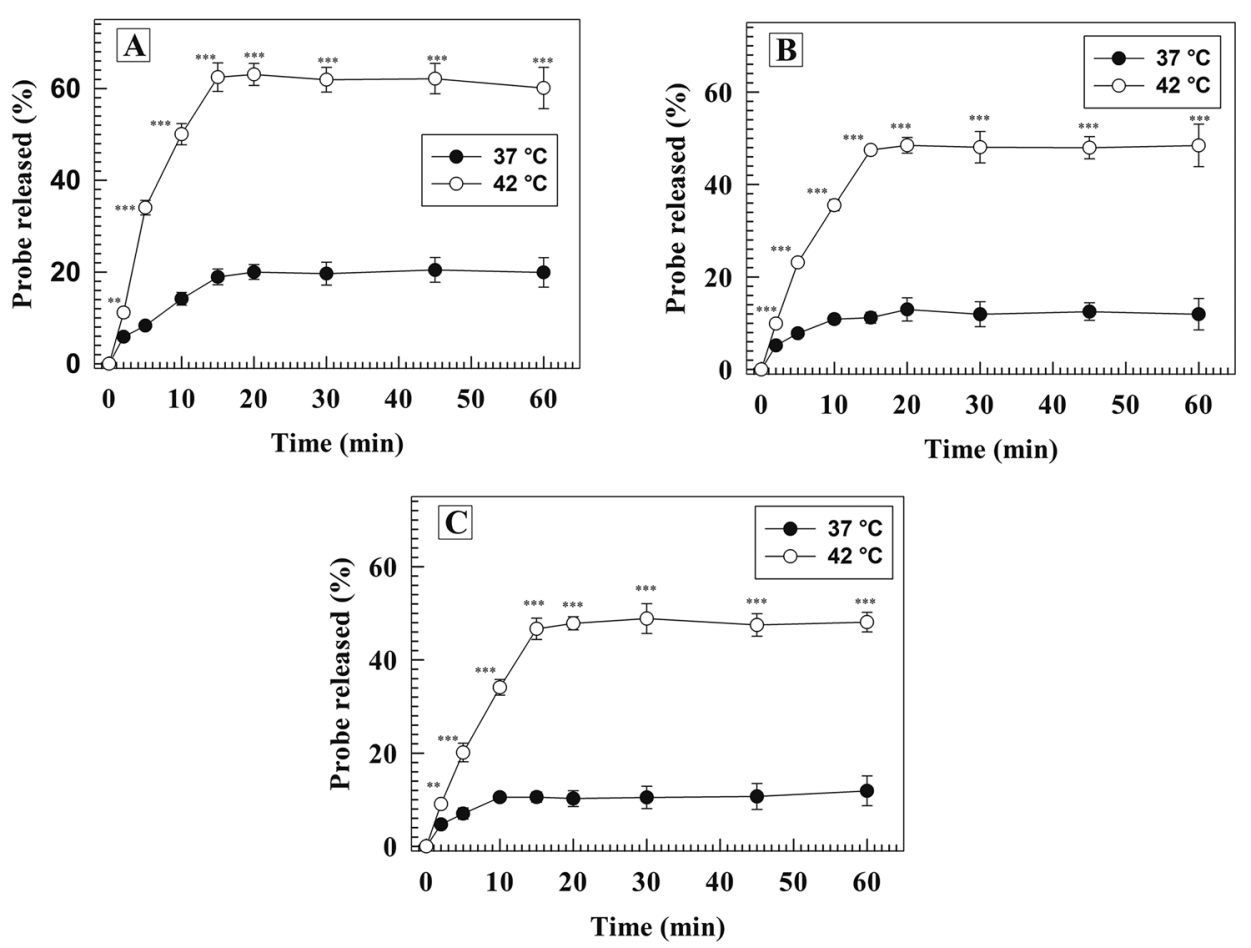
Release studies. Kinetic release profiles of fluorescent probe from ThermoLipo (**A**), hybrid M0 (**B**), and hybrid M1 (**C**) at 37 °C and 42 °C in PBS buffer (0.01 M, pH 7.4). Results are the mean of three independent analyses ± S.D

The validation of the thermoresponsiveness of both liposomes and hybrid systems was assessed by evaluating the release profiles of a hydrophilic fluorescent probe at 37 °C and 42 °C, which reflect body temperature and the hyperthermia state, respectively. As Fig. 6 shows, for each time point, many significant differences between the two investigated temperatures. In particular, less than 20% of fluorescent probe was released from ThermoLipo in 60 min, while the release rate was around 60% for the same formulation under mild hyperthermia (Fig. 6A). The thermoresponsiveness was also recorded for hybrid nanovesicles, confirming the happened membranes’ fusion and the keeping of ThermoLipo-derived features (Fig. 6B and C).

Despite the kinetic release profile of hybrids seems to be so close to the liposomes’ one, focusing on the liposomes’ release amounts (Fig. 6A), the percentage is higher in absolute values. Probably, this could be due to the presence of cholesterol, derived from the EVs’ bilayers after the hybridization process, which led to a slight increase in the rigidity of the resulting nanosystems [65]. These results were also in line with data obtained by TSI investigation through Turbiscan Lab above described (Fig. S4, ESM_1).

### In vivo tumor targeting studies

One of the main challenges of anticancer nanomedicine is to provide drug delivery systems able to accumulate in target sites, avoiding the body spreading, thus improving the efficacy of payloads and reduce at the same time the side effects on healthy tissues [66–69]. For this reason, in the last two decades, tons of studies explored the potential use of targeting molecules to decorate the surface of nanocarriers in order to obtain a site-specific nanomedicine [70, 71]. However, despite the in vitro and in vivo results being very encouraging in small animal models, often these sophisticated approaches fail in clinical practise. One of the main reasons for these failures can be attributed to the formation of protein corona around the nanosystems after injection, which makes the target properties, providing a new “biological identity” to the nanocarriers [72]. A potential strategy to overcome these limitations may be obtained exploiting the intrinsic capability of some cells, such as monocytes, to migrate toward specific body sites in response to altered signals. In these attempts, monocyte- or leukocyte-derived vesicles have been used as potential nanomedicine for cancer targeting [58]. Recently, M1-macrophage-derived EVs were also investigated as a potential treatment for cancer, thus showing suitable targeting properties and the ability to improve the efficacy of payloads by promoting the shift of TAMs from M2 to M1 phenotype [41, 73].

**Fig. 7:**
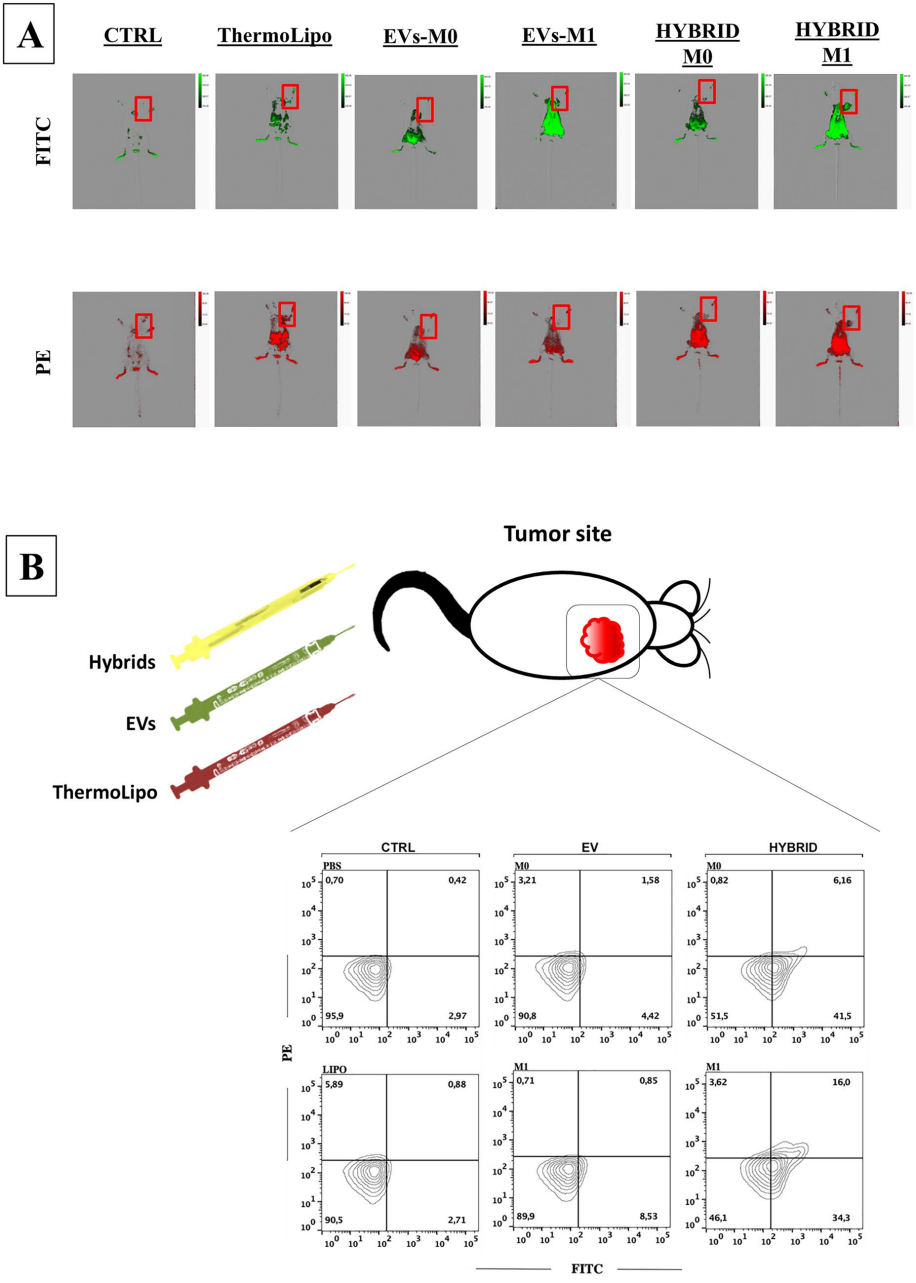
In vivo tumor targeting analyses. The upper part of the panel showed in vivo live imaging studies 3 h after the injection of hybrid nanovesicles, while the bottom part reports ex vivo cytofluorimetric analysis on tumor tissues 3 h post sample injection. Flow cytometry plots show the percentage of double-negative tumor site-derived cells after PBS injection (negative control); PE-positive cells after rhodamine-labeled liposomes (ThermoLipo control); FITC-positive cells after CFSE-stained EVs (macrophage-derived EVs controls); double-positive cells after hybrid systems M0 and M1. Results are the mean of three independent analyses and are representative of all involved animals (*n* = 3)

Distribution profiles were investigated up to 3 h, and then the animals were sacrificed. In particular, the accumulation rate in tumor tissues was very low after 1 h, while it appears significant after 3 h (Fig. 7 and Fig. S5, ESM_1). As shown in Fig. 7, M1-EVs showed an almost double accumulation in tumor tissue compared to M0-EVs (4.4% vs 8.5%, respectively). Interestingly, the hybridization process improves the ability of resulting nanocarriers to accumulate in TME. Although these findings are not completely understood and need more investigation to be clarified, they can be considered in line with data obtained by the TSI investigation. Indeed, the improved stability of hybrid systems compared to native EVs may lead to a higher stability also in biological fluids, resulting in a higher tumor accumulation extent. In particular, the best results were obtained by employing hybrids M1 that show an accumulation rate twice as much as EVs-M1. Moreover, both hybrid nanovesicles showed an accumulation higher than liposomes.

These preliminary results suggest a potential use of hybrid M1 systems as a nanoplatform for the targeted delivery in solid tumor treatment. Moreover, the natural cargos of EVs-M1 and the acquired thermoresponsiveness may strongly improve the therapeutic efficacy of payloads, thus providing synergistic effects of both liposomes and EVs-M1 features.

## Conclusions and future perspectives

In this study, hybrid nanoformulations composed by ThermoLiposomes and macrophage-derived extracellular vesicles have been investigated in order to develop a nanoplatform for a potential immuno-chemotherapeutic treatment. This aim develops out of the need to overcome all the limitations of the current and common anticancer therapies as well as to exploit the developments in the field of extracellular vesicles. In particular, we combined the targeting of the tumor site with the improvement of the systems’ release rate by using external stimulus like induced hyperthermia, in the form of a single approach. Indeed, all the formulations used have been investigated in terms of physicochemical features, thus confirming the presence of optimized resulting hybrids. Moreover, the happened fusion has been verified through FACS analysis, which further demonstrated that the freeze and thaw method used did not affect the bilayer structures, especially the surface proteins displayed. The thermoresponsiveness derived from the low transition temperature liposomes has also been reported from the fused nanovesicles, which through in vitro studies of a fluorescent probe showed release profiles trends similar to the native synthetic liposomes. Finally, the tumor targeting studies have demonstrated the suitability of these hybrid nanovesicles when administered to mice in reaching the B16F10 cell-induced tumor site in the early hours after i.v. infusion, when compared to the liposomes control; data were confirmed both from animal live imaging performed at 1 h and 3 h and from tumor tissue cytofluorimetric analysis at 3 h. In particular, the highest tumor accumulation rate was obtained by using hybrid M1. These results confirmed the ability of this nanosystem to combine the advantages of both nanotechnologies and highlight their potential use for realizing an effective and safe personalized anticancer nanomedicine. Despite clinical translation is limited in terms of scale-up protocols set for EVs’ isolation, as well as low-yield engineering strategies [74], the role of immune cells-derived EVs into precision medicine can be developed as a future treatment of several pathologies. In particular, the involvement of the immune system in the treatment of cancer is already well-known, thus the opportunity to isolate monocytes from the peripheral blood of patients, differentiate them into M1 phenotype, also exploiting as EVs’ source [75] could be a turning point in the oncological field.

### Supplementary Information

Below is the link to the electronic supplementary material.

Supplementary file1 (DOCX 977 KB)

## Data Availability

The datasets generated during and/or analyzed during the current study are available from the corresponding author on reasonable request.
