# Supplementary material for: Thermoresponsive M1 macrophage-derived hybrid nanovesicles for improved in vivo tumor targeting
Source: Drug Deliv Transl Res. 2023 Jun 26;13(12):3154–68. doi: 10.1007/s13346-023-01378-9 (PMC10624726; doi:10.1007/s13346-023-01378-9)
Supplement: Supplementary file 1 — Supplementary file1 (DOCX 977 KB) [file 13346_2023_1378_MOESM1_ESM.docx]

**Supplementary materials**

**Thermoresponsive M1 macrophage-derived hybrid nanovesicles for improved in vivo tumor targeting**

Antonella Barone^1^, Anna Maria Zimbo^1^, Nicola d’Avanzo^1^, Anna Maria Tolomeo^2^, Stefano Ruga^3^, Antonio Cardamone^3^, Christian Celia^4, 5, 6^, Mariangela Scalise^1^, Daniele Torella^1^, Massimo La Deda ^7,8^, Enrico Iaccino^1*^, Donatella Paolino^1*^

^1^ Department of Experimental and Clinical Medicine, University "Magna Græcia" of Catanzaro Campus Universitario-Germaneto, Viale Europa, 88100 Catanzaro, Italy.

^2^ Department of Cardiac, Thoracic and Vascular Science and Public Health, University of Padova, 35128 Padua, Italy

^3^ Pharmacology Laboratory, Institute of Research for Food Safety and Health IRC-FSH, Department of Health Sciences, University Magna Graecia of Catanzaro, 88100, Catanzaro, Italy

^4^Department of Pharmacy, University of Chieti – Pescara “G. d’Annunzio”, 66100 Chieti, Italy

^5^Lithuanian University of Health Sciences, Laboratory of Drug Targets Histopathology, Institute of Cardiology, A. Mickeviciaus g. 9, LT-44307 Kaunas, Lithuania

^6^Institute of Nanochemistry and Nanobiology, School of Environmental and Chemical Engineering,

Shanghai University, Shanghai 200444, China

^7^Department of Chemistry and Chemical Technologies, University of Calabria, 87036 Rende, Italy

^8^CNR-NANOTEC, Institute of Nanotechnology U.O.S, Cosenza, 87036 Rende, Italy

*corresponding author: [iaccino@unicz.it](mailto:iaccino@unicz.it); [paolino@unicz.it](mailto:paolino@unicz.it)


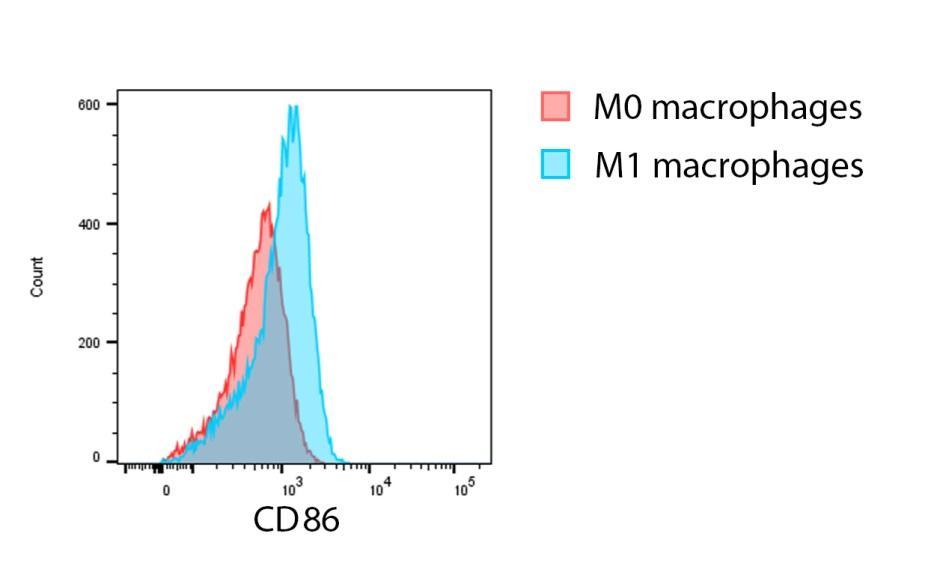


**Fig. S1** Expression CD86 marker on untreated and M1 polarized J774A.1 cells

**Table S1**. Colloidal properties of fluorescein-loaded ThermoLipo and CFSE-EVs.

| **Samples** | **Size (nm)** | **ξ (mV)** | **PdI** |
| --- | --- | --- | --- |
| Fluorescein-loaded ThermoLipo | 121 ± 2 | -13.6 ± 0.7 | 0.06 ± 0.02 |
| CFSE-EVs M0 | 158 ± 11 | -4.66 ± 0.36 | 0.24 ± 0.07 |
| CFSE-EVs M1 | 169 ± 4 | -14 ± 2.6 | 0.27 ± 0.02 |

Footnotes: ξ: zeta potential; PdI: polydispersity index.


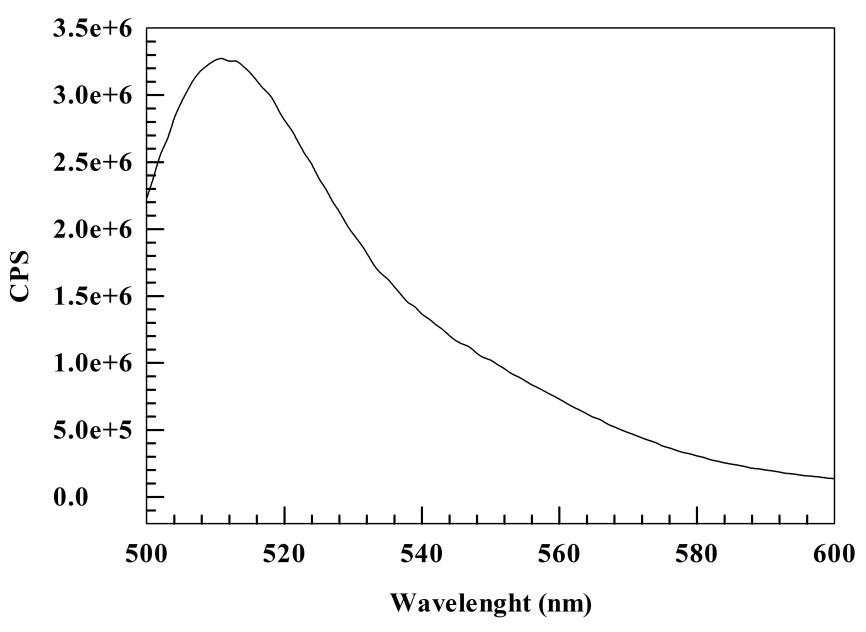


**Fig. S2.** Emission spectrum of fluorescein-loaded liposomes. Fluorescein-loaded liposomes were excited at λ_ex_ = 490, then the emission spectrum was recorded in a wavelength range between 500 and 600 nm. Results are representative of three independent analyses.


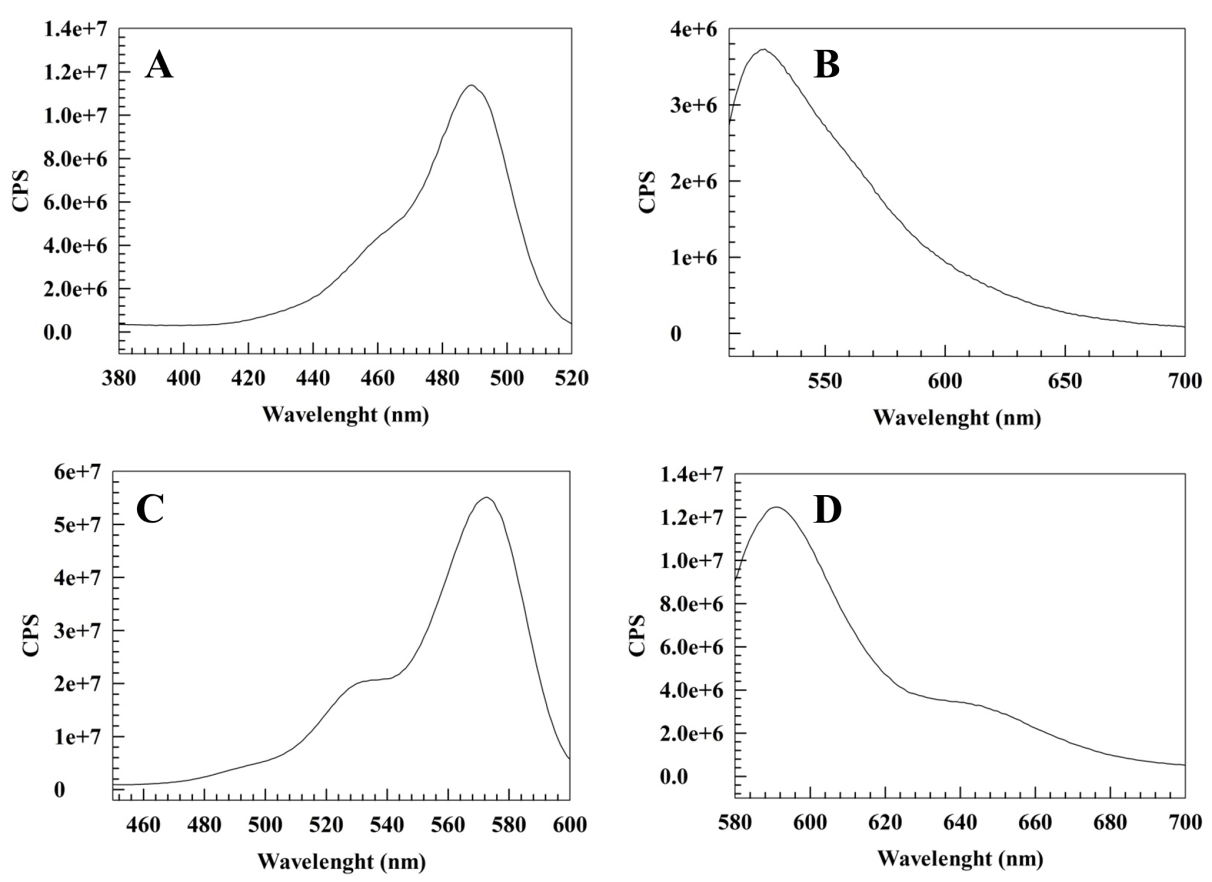


**Fig.S3**. Excitation/emission spectra of CFSE-EVs and Rho-Lipo. (A). Excitation spectrum of CFSE-EVs (λ_em_ = 525 nm); (B). Emission spectrum of CFSE-EVs (λ_ex_ = 490 nm); (C). Excitation spectrum of Rho-Lipo (λ_em_ = 605 nm); (D). Emission spectrum of Rho-Lipo (λ_ex_ = 570 nm)


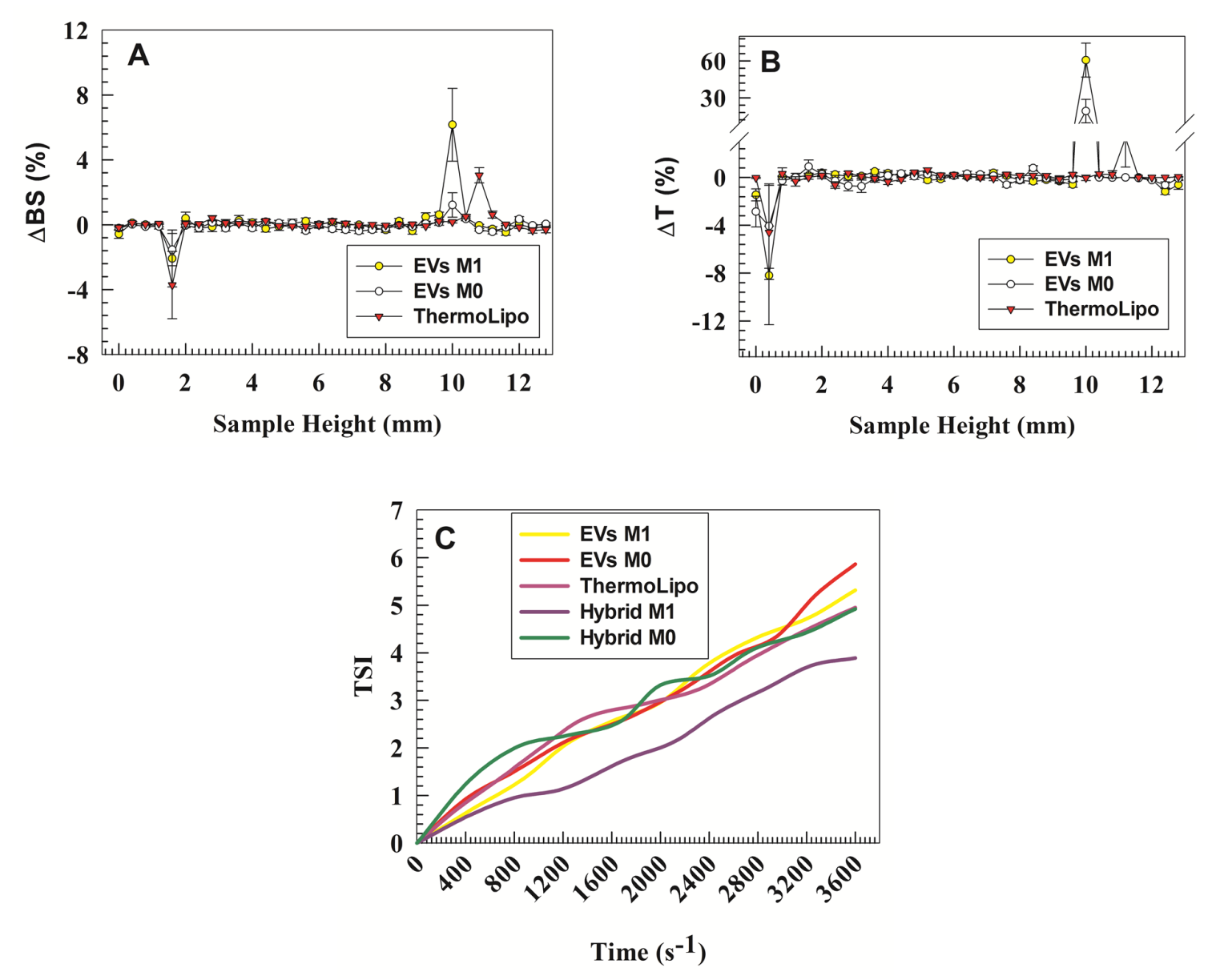


# Fig. S4 Turbiscan analysis of ThermoLipo, EVs-M0 and EVs-M1. Analyses have been carried out at 25 °C for 1 h. (a) Δ Back Scattering; (b) Δ Trasmittance and (c) TSI are the mean of three independent analysis ± S.D


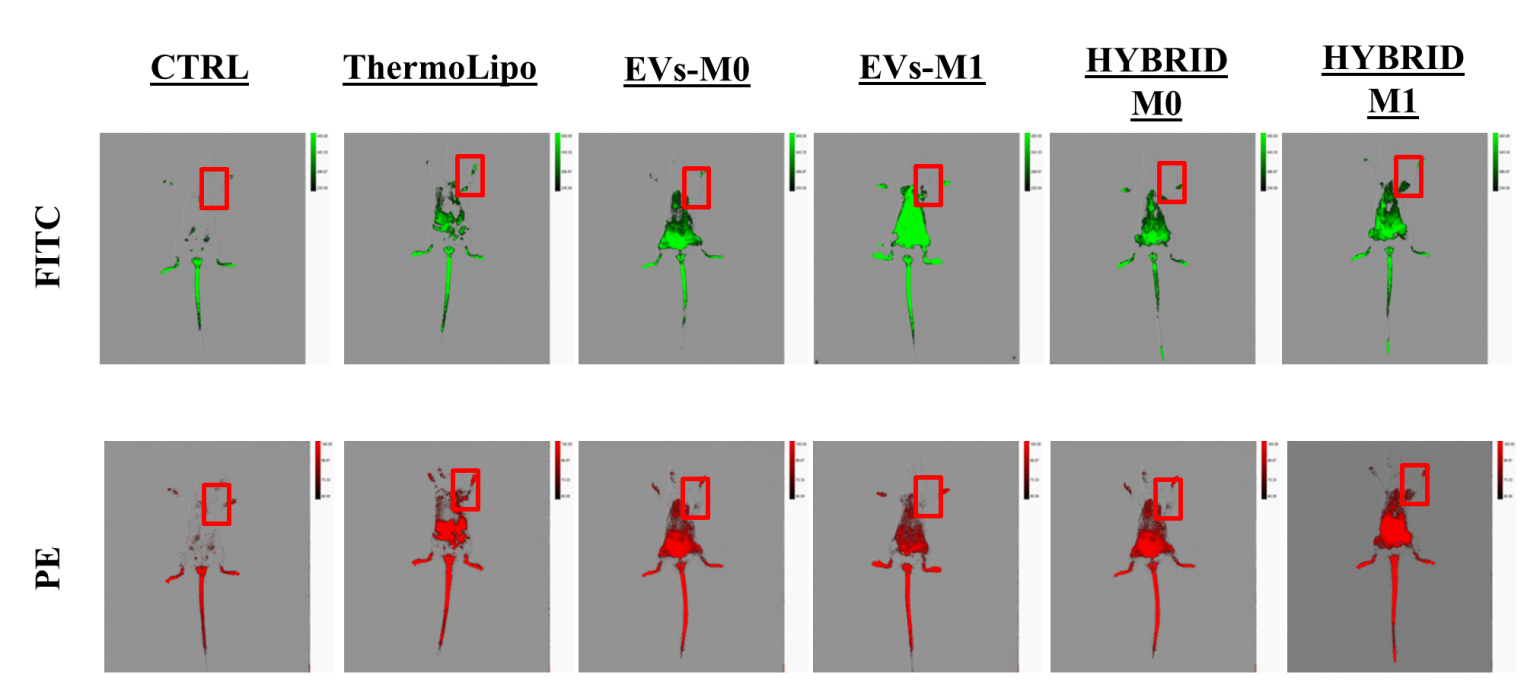


**Fig. S5** *In vivo* live imaging analysis of ThermoLipo, EVs and Hybrid nanosystems. Analysis was performed 1 hour after administration
